# Hospital and Institutional News

**Published:** 1917-03-31

**Authors:** 


### HOSPITAL AND INSTITUTIONAL NEWS.

### A NEW DEPARTURE AT THE MIDDLESEX HOSPITAL.

According to an obviously inspired article by the medical correspondent of the *Times*, the authorities of the Middlesex Hospital are about to embark on a great extension of the laboratory accommodation for research work into the causation and treatment of serious diseases. This will, presumably, be largely by an augmentation of the already existing Bland-Sutton Institute of Pathology attached to the hospital. It is also stated that "within the next few days" the public will be taken fully into confidence and told the story of the work already accomplished there. The article proceeds to a eulogy of research in general; and to the expression of a belief, which is probably quite accurate, that the public are growing ever more anxious to help the cause of scientific progress, in spite of the activities of anti-societies of various kinds. It is matter for rejoicing that this should be the case; much of the credit for it belongs to the late Lord Cromer and to Mr. Stephen Paget, for their labours of love on behalf of the Research Defence Society. At the same time, there is just one note of warning to be struck: it is so easy to "eyewash" the public, because of the latter's profound ignorance of science and scientific methods, that it is of the very utmost importance that all hospitals promoting schemes for research should sedulously avoid even the appearance of playing to the gallery when publishing their plans for the future and their records of the past.

### MILITARY HOSPITAL EXPANSION IN WALES.

Under the supervision of the Red Cross authorities many new hospitals are being opened in South Wales, for the demands of the military have outgrown the accommodation available. The Carmarthen Guardians have placed at the Society's disposal the entire new block of buildings at the workhouse, including the kitchen and adjoining rooms. This will, of course, result in some dislocation of the board's arrangements, but the casual ward is to be closed, and in the event of the accommodation thus liberated being insufficient a certain number of the inmates will be transferred to one of the neighbouring workhouses. The chief difficulty has been to provide for the care of the aged bed-ridden inmates. The Carmarthen Red Cross Society received the offer of several country mansions for use as hospitals, but these were found much less suitable than the workhouse. The borough of Swansea has been asked to provide 200 additional beds immediately, and the Education Committee, in response to this urgent request, has handed over Brynmill Infants' School. It will cost £270 to alter the premises for the purpose of a hospital and restore it afterwards, and this sum, it is understood, will be forthcoming either from the War Office or the Red Cross Society. There was no hesitation on the part of the Swansea people in transferring the school, the Mayor declaring that he was prepared to give everything that was wanted—even if that meant all the schools in the borough! The Bridgend infirmary and maternity wards have been placed at the disposal of the military authorities by the Board of Guardians. Accommodation will thus be provided for 120 new beds. The hospital will still be conducted by the board, subject to the military authorities agreeing to employ an adequate staff—the continuance of the existing staff is recommended—and to pay all the expenses connected with the institution except the loan charges. Under the arrangement the Guardians, while they will make no profit, will be protected against any loss.

### CONSTITUTIONAL CHANGES AT THE ROYAL SUSSEX COUNTY HOSPITAL.

Following on the correspondence in the local press (commented on in The Hospital, March 17, 1917, p. 473) as to the election of women to the board of the Royal Sussex County Hospital, notice has now been given that a Special General Court of Governors will be held at the hospital on April 4, on the requisition of twelve Governors. A Governor will move that Statutes 33 and 48 be amended by the deletion of the word "male" in them, thus entitling women to be members of the board of management. Another Governor has given notice of an amendment to this motion, which concurs in the alteration of Statute 33 but refers to the board of management the "question of the alteration of Statute 48, together with the whole question of the constitution of the board of management, the proportion of women representatives upon it, and the consequent necessary amendment of the Statutes," with a view to the decision of all these points by the Annual General Court next year. At all events, these motions should provide a lively debate, and institutional managers will look forward to the report of it with unusual interest.

### RESIDENT MEDICAL OFFICERS AT PRIVATE WAR HOSPITALS.

On a previous occasion The Hospital has entered a protest against the wasteful policy of employing a resident medical officer at small private war hospitals, at any rate when they are situated in large towns. Given the latter condition, it is perfectly possible for a non-resident part-time medical officer to run a primary hospital (one, that is, receiving wounded patients straight from the hospital ships) of a hundred beds without the slightest detriment to the welfare of a single patient. The deduction would seem to follow that for a hospital of less size to attempt to employ the whole-time services of a resident medical officer is a gross waste of medical service and (if he is salaried) of money too. Within the last few days a case has come to our ears in which a practising London surgeon was asked to become resident medical officer at a private military hospital of under thirty beds—an offer which was very properly at once refused. To judge by newspaper reports, the new American Hospital for English Officers recently opened at Lancaster Gate will also employ a resident for the forty-five beds which the hospital provides. In this case the resident is, we suppose, an American doctor, for whose services to our cause we should be and are duly thankful. All the same, his sacrifice of his time and his home ties on our behalf could be made more fruitful to the Allied cause if he were employed in a part-time capacity at that hospital; for we make bold to say that his duties will not take up more of his time on the average than two, or at the most three, hours a day. When a convalescing or invalided medical officer is employed in this capacity of resident at a private hospital, as a form of light work, there is, of course, no objection to be urged. Save for that exception, our former protest is emphatically renewed.

### PROGRESS OF THE LEICESTER SATURDAY HOSPITAL SOCIETY.

Not the least interesting feature of the annual meeting of the above Society, held in the out-patients' department of the infirmary, was an intimation by Mr. J. Gipson Clarke (who presided) that as soon as the war is over and the entire position of the Society can be reviewed, the committee will take into consideration the acquisition of a Children's Home. The progress of this Society has been remarkable. Founded some fifteen years ago under the enthusiastic lead of Sir Edward Wood, the Society acquired its first home (the Desford Hall Convalescent Home for Men) as its starting point. Some ten years afterwards the Swithland Home for Women was built, and such is the popularity of the movement that it seems likely before the Society reaches its twentieth year a Children's Home will be founded. The report for the year showed that the contributions totalled £16,450, an increase of £540 on the figures of 1915; 408 females and 385 males were received into the Society's homes and other institutions during the year. The Desford Hall Home was placed at the disposal of the military authorities for convalescent soldiers, of whom 339 were received. The Society's invested capital is £6,860, with £639 on bank deposit and £400 in hand. The report was supported by interesting speeches from Mr. S. A. Gimson (vice-president), the Mayor of Leicester (Alderman J. North), Mr. T. Fielding Johnson, jun. (chairman of the board), Mr. B. W. Russell (chairman of the house committee of the infirmary), and Major Blakesley, and was adopted unanimously. Sir Edward Wood was re-elected president, Mr. J. Gipson Clarke and Mr. S. A. Gimson (vice-presidents), and Mr. Alfred Corah, J.P., treasurer in the place of his brother, the late Mr. J. A. Corah.

### ST. BARTHOLOMEW'S HOSPITAL AND THE VENEREAL CRUSADE.

It has been pointed out that a sentence in our issue for March 17, 1917, p. 477, may be read as meaning that the management of St. Bartholomew's Hospital has been backward in their support of the concerted schemes for curing and preventing venereal diseases now being organised under the auspices of the Local Government Board and of local authorities. The sentence in question stated, quite correctly, that all the big teaching hospitals except St. Bartholomew's are represented in the London County scheme for the establishment of clinics. The reason why St. Bartholomew's is not represented is, of course, that this hospital is in the City of London and has undertaken to work a scheme under the Corporation, as described in one of our former issues (The Hospital, February 3, 1917, p. 367). That the wording of our article on p. 477 should have been capable of being misinterpreted into an insinuation against the management of the doyen of London hospitals we much regret. For a description of the excellent work and arrangements at St. Bartholomew's in this connection, our article of February 3 should be consulted, as it amply serves to show what is being done and how thoroughly the problem is being tackled.

### HOSPITAL MEDICAL OFFICERS AND DYING DECLARATIONS.

A recent case at the York Assizes, in which the judge ruled against the admission of a "dying declaration" as evidence and the prosecution thereupon submitted to a verdict of "Not guilty" concerning the indicted practitioner, and another case which occurred in London only three or four months ago, are of some importance to hospital residents, chaplains, and visiting staffs. In the case tried at York the successful objection was based mainly on the facts that the minister who took the declaration asked questions of the woman concerned; and that she did not in fact die until nine or ten days afterwards, and was therefore not dying at the time her evidence was taken. It might some day turn out a nice legal point as to where precisely the line is to be drawn to constitute a patient, who subsequently dies, a dying person at any precise period of time beforehand, in the eyes of the law. Obviously, a medical man who has charge of such a patient must bear this point in mind in deciding whether to allow the deposition to be taken: if he waits too long, the patient may die or lose consciousness; if he does not wait long enough, the evidence may be rejected on the ground that the patient was not dying when it was given. Every doctor knows, or ought to know, that for such a "dying declaration" to be admissible afterwards as evidence in a Court of law, the patient must not only be dying, but must herself be firmly convinced that she is dying and has no hope of recovery. The London case, to which reference has already been made, brought out a different point, which so far as we know has not been raised before; and inasmuch as the case never got as far as a magistrate's court, it may be worth while to mention certain details of it.

### THE DOCTOR'S RESPONSIBILITY IN THESE CASES.

A patient was admitted to a London hospital in a state of grave abdominal sepsis, due fairly clearly to a criminally produced abortion. In consequence of certain information received by the police (before her admission to hospital, if we remember rightly) a detective called at the hospital, accompanied by a magistrate, to secure a dying declaration as to the guilt of certain suspected persons. A visiting surgeon of experience and strength of mind, in whose ward the patient was under treatment, happened to meet the detective and the magistrate just as they were about to enter the ward. On learning their business he promptly declined to allow the deposition to be taken. His grounds for refusal were that, to begin with, he was not then certain that the patient would die; and to go on with, that the fright of being subjected to the formalities of a dying declaration would deprive her of such chance of life as she still had. In the event, the patient lived for another week and more, thus justifying the prognosis of the surgeon that the case was not at the time entirely hopeless; and the dying declaration was never taken. It is certainly a point for doctors and lawyers to remember that a patient whose condition seems desperate might be unfavourably influenced by being told that her death is imminent and deprived thereby of the last flicker of a chance of life. Yet a further point in the same connection was alleged in the York case; this was that although the woman told the minister who took her statement she was dying, she had been told by a doctor that she was not. Inasmuch as the state of a patient with septic peritonitis often changes very rapidly, it does not seem as if much importance should be attached to this particular type of objection so long as the patient is clearly under the belief, when giving her evidence, that death is inevitable.

### THE LIVERPOOL HAHNEMANN HOSPITAL.

Many points for congratulation were mentioned at the annual meeting of the Hahnemann Hospital, Liverpool. The story of the institution's good work during the year, as told by the honorary secretary, Mr. Thomas Cooper, was reinforced by a personal tribute from the Rev. J. McKinney, who testified to the very great skill which he had experienced in the medical treatment of his family during serious illness. The report of the honorary treasurer, Mr. Harold G. Crosfield, was of the variety that causes no anxiety, for he was able to announce a balance of £805 on the right side, a happy result partly accounted for by the fact that the hospital had a regular income of £2,334. In response to an urgent request, the number of beds set apart for military patients has been increased to fifty-four, which is the utmost accommodation the institution is able to allocate. The Lord Mayor, who presided, congratulated the Hahnemann Hospital and all those connected with it on what had been accomplished, and offered his congratulations also to Miss Tank-Davis, the lady-superintendent, on receiving the honour of the Royal Red Cross, First Class.

### THE DEEDS OF SELECTION COMMITTEES.

A remarkable correspondence has appeared in the Plymouth papers, in which the doings of the Australian Branch of the British Red Cross Society in Plymouth have been subjected to criticism. As the chief critic is herself, it appears, an Australian, we are not arousing Imperial dissension in alluding to the allegations which she has made. She declares that her request to be put upon the official visiting list of those authorised to distribute comforts to the numerous Australian wounded in Plymouth was declined; that her gifts of the scarce, but naturally popular, Australian newspapers were refused; that the "carefully selected" list of visitors included other than Australians; and that, in short, she was snubbed all round. Such categorical charges can hardly be entirely destitute of truth, and, taking them at their face value, we ask, as impartial observers, whether any sensible committee having to do with hospital affairs would so discourage a prospective helper and her gifts as seems to have been the case at Plymouth. It is the point of honour of every sensible hospital committee not to snub anyone, and that, with whatever reason, the lady in question has been snubbed is at all events clear. The phrase quoted, "carefully selected ladies," has itself an unfortunate sound, and, since the lady has shown herself well able to defend herself, we note merely how much harm a hospital committee may do which, with whatever motive, resorts to snubbing.

### THE ETHICS OF HOSPITAL DINNERS IN WAR-TIME.

An informal discussion took place the other day as to the advisability of holding a festival war menu dinner this year in aid of the funds of a certain hospital. The following are some of the arguments used in favour of such a scheme, and some of the arguments used against it. The arguments for holding the dinner were that four or five thousand pounds would be thrown away if it were not held; investments ought to be increased, and the best way of doing that is to increase the income; that every such public function makes more new friends and more "new" money; that a voluntary charity must advertise; that, if the war continues, it is possible that the office staff next year will be depleted, and it will be more difficult to organise a dinner. On the other hand, the arguments against were a fear lest the hospital should be charged with encouraging the consumption of unnecessary food, or of spending money on paper and wasting printers' labour; that the sum so raised "could be put towards winning the war," or, in other words (astounding statement!), spent more usefully; that the hospital could really afford to do without it; that unthinking people regard a dinner as an extravagant jollification; and, lastly, that many supporters are taught to regard the need for a dinner appeal as an actual defect in management. This is a curious collection of pros and, especially, cons. In the end the discussion of the whole subject was deferred for a month.

### A DEVONSHIRE HOSPITALS FUND.

The following grants to hospitals and kindred institutions in Devonshire were made recently at a meeting in Exeter of the executive committee of the Queen Victoria Commemoration and King Edward VII. Memorial Funds:—Royal Devon and Exeter Hospital £130, Devon County Nursing Association £75, Torbay Infirmary £37 10s., North Devon Infirmary £30, Royal Albert Hospital £25, Exeter Nursing Association £21, Bideford Hospital £13 13s., Tiverton Hospital £13 13s., Torquay Nursing Association £12 10s., Ockenden Convalescent Home £10 10s., Ashburton Cottage Hospital £9, and Ottery Cottage Hospital £9.

### THE NEEDS OF THE CIVIL POPULATION.

In these days, when compulsion for doctors and the needs of the Army are in everybody's mouth, it is well to record that the needs of civilians are, in the long run, no less important. For instance, the following announcement has been published and is worth a moment's thought: "There is only one doctor left to the 13,000 people of Shirebrook, a Derbyshire mining town," states the *Daily Telegraph*, "where a serious epidemic of scarlet fever has broken out." Comment is needless. To leave towns exposed to the risk of epidemics without doctors is not good sense, nor statesmanship, nor organisation.

### "HAVE YOU ANY POTATOES?"

Food difficulties enter so much into our daily thoughts and conversation that at certain times and seasons it would be a relief if an arrangement could be made to consider the subject as taboo. But those who have to arrange for the feeding of a large number of fellow-creatures in a hospital or institution must be excused if they try to pick up useful information at a fitting opportunity. "Have you any potatoes?" is what one hospital secretary generally asks another when they meet. The answer is rarely in the affirmative, and the conversation drifts to butter beans and swedes. The bean, whether butter or haricot, is met with so frequently nowadays that it is building up for itself a real danger of a prolonged period of estrangement after the war, when the Briton will return to the potato that he never realised he liked so much. And then, when we unwillingly eat of our beans, rice, turnips, and unaccustomed flesh-pots we do so with the additional irritation that weight for weight many of these foods are four times the price of potatoes. In this respect the cereals have increased in price much more than the perishable vegetables, although, as a profiteer, the wholesale grocer is regarded with less suspicion than the farmer.

### A STAFF AND SOLDIERS AT THE SPADE.

The scarcity of potatoes, which we fear there is some reason to believe is artificially increased through the greed of those growers who are holding back stocks for a further rise in price, lends interest to any serious attempt on the part of hospitals to increase their area of potato cultivation. At the Coventry and Warwickshire Hospital, for instance, the nurses and the more able of the wounded soldiers have turned over a patch of ground measuring 100 ft. by 30 ft. This land forms part of the old Corporation drying-ground, and there is another patch available at the end of the hospital garden. Since the difficulty in many cases has been as much want of labour as want of land, these volunteers deserve praise; but though digging is an art, and a strenuous one, it can be learnt with a little skilled supervision, and accomplished by regular shifts adapted to the strength of even weakly people and women.

### THE HOSPITAL STANDARD AND THE DOMESTIC STAFF.

It is now a long time since we ventured the prophecy that the improvement in the conditions of hospital nurses would soon be followed by a similar improvement in the accommodation at present afforded to the domestic staff. The separate bedroom, the proper relaxation-room, even perhaps separate quarters analogous to the Nurses' Home may yet be recognised as necessary, and in many cases the need for a much higher standard is as great as it is often neglected. We are glad to notice every sign of improvement in this direction, and would therefore draw attention to an interesting building scheme which has been successfully started by Mr. C. E. Ridley, with the help of Mr. Courtauld, the High Sheriff, in connection with the Chelmsford and Essex Hospital. This old friend of the hospital voiced last year the need for better accommodation for the domestic officers and the staff at the institution. But he has not rested there. Since June of last year, as a result of a series of personal letters, Mr. Ridley has raised a sum of about £2,500, to which he has contributed £500 himself. He estimates that £4,000 will be required. This would enable the kitchen to be enlarged, a servants' hall to be provided, and more bedrooms to be built. We trust that these will include a separate bedroom for each of the domestic staff, and that the proposed servants' hall is a proof that their comfort as well as an improved kitchen will be provided for. It is necessary to emphasise the need, since only a year or two ago we were shocked at the dark and almost subterranean quarters allotted to the staff in the chief hospital of a Midland city. The rooms were like a series of caverns, in which we were surprised that any servants consented to live.

### RICE FLOUR AND MAIZE FLOUR FOR BREAD.

The Keighley Board of Guardians has been investigating the problem of food supplies, and the master has informed the board that he has experimented with sample loaves, containing 50 per cent. of rice flour and 30 per cent. of maize flour, and that it appeared that, if either of these substitutes were permitted, the flour allowed might be brought within the official scale. This is interesting, as the master has found it impossible to reduce the quantity of bread to the official allowance, though he has complied with it so far as meat and sugar are concerned. An experiment on the lines of his suggestion with regard to bread has been decided on, and after a week's trial the report is awaited.

### THE KILLING OF ASYLUM COWS.

The committee of the Fulbourn Asylum is anxious to act patriotically in regard to the management of its cows, and complains that the School of Agriculture at Cambridge has not satisfactorily answered their question, which was: "Are we acting in the best interests of the country in killing our cows after the period of lactation is over?" Whether they are so acting is not yet clear to themselves, though they have no doubt that the practice pays them economically. The proposal to pursue inquiries to the War Agricultural Committee was dropped. The cows are said to bring in £100 a year, and as the committee appears to include three practical farmers, presumably they are well advised.

### HOSPITALS SHAREHOLDERS IN DRAPERY ESTABLISHMENT.

Under the will of the late Mr. Frank Debenham, of Messrs. Debenham and Freebody, Middlesex Hospital and Guy's Hospital become shareholders in the great West-end drapery business. Each of these hospitals will become possessed of thirty £6 per cent. preferred ordinary shares, and the Orphan School at Watford will receive also twenty shares. The Middlesex Hospital will eventually benefit further under the will, as after the decease of Mr. Debenham's three daughters certain leasehold property in Mortimer Street will pass to that institution. Mr. Frank Debenham has been a consistent and generous subscriber to London charities for many years, and philanthropy and public spirit are further marked by the very thoughtful provision under which his executors are authorised to continue for three years his usual subscriptions to hospitals, schools, and charitable and benevolent institutions. We think we are right in saying that some years ago Mr. Debenham was a prime mover in the formation of a company whose dividends were to be limited to 4 per cent. and any surplus profits to be used to cheapen the price of the goods dealt in, which were supplies of instruments, dressings, and other surgical requisites to hospitals. We do not know if the results of the trading brought about the object intended—namely, to assist the medical charities to obtain supplies in the most favourable market.

### THE HOUR OF THE ANNUAL MEETING.

Two speakers at the recent annual meeting of the Royal Aberdeen Hospital for Sick Children criticised the time of day at which it was held, on the ground that the hour prevented the attendance of working-class people who would otherwise be interested and anxious to help the hospital. Their criticism was unexpectedly endorsed by the chairman, Sir Thomas Burnett, who said that he had to waste a whole day in order to attend, and that the hour was inconvenient to those who lived in the country. Another hour was suggested but not decided on, but a convenient hour and, be it added, a convenient day are precisely two of those humble details on which the success of a hospital depends. We hope a different and convenient day and hour may soon be announced, and that this will be the first step in a greater effort to enlist the sympathy of the large working-class population of the city.

### MUNITION GIRLS AND THE WOUNDED.

The initiative of half a dozen girls in Messrs. Greenwood and Batley's munition factory has gained a really remarkable success. The friendly six wished to add to the comfort of the wounded at Allerton House Hospital, Yorkshire, and began by selling scented cards at a penny each. When the matron informed them that the men wanted a billiard table, they were not at all overwhelmed at the task of raising the sum necessary to pay for it, but, enlisting the co-operation of the other workpeople, collected £45 and bought the table in very quick time. This success led to the formation of a workpeople committee; voluntary collections were held every week, and £500 has now been raised by these means. Their gifts include the billiard table, two pianos, two gramophones with fifty records, a collection of games, thirty counterpanes, and four bed-screens to Armley Hospital, and 122 pairs of slippers for the wounded at Leeds General Infirmary. This is not all, and money is still in hand. Of course, without help this fine result could not have been attained, but the six girls deserve public thanks for giving the start, and we hope that the matron of the Allerton House Hospital, the soldiers there, and Mr. Hodgson, the secretary of the Soldiers' Comforts Fund in the factory where the girls work, will see that they get it.

### NURSES' UNIFORMS FOR HEALTH VISITORS.

Bethnal Green Borough Council's Health Committee has been considering the question of the provision for the health visitors of uniform clothing of the style usually worn by nurses, and is of opinion that such distinctive dress is very desirable in view of the character of the duties carried out by the health visitors. The committee feels that uniform is calculated to support the position of these officers and to be in every way more suitable to the character of the work they perform than ordinary civilian dress. The cost of the proposed uniform clothing will be approximately £5 5s. in respect of each health visitor. The committee recommends that each health visitor in the service of the Council be provided with, and be required to wear during usual hours of duty, uniform clothing of the style usually worn by nurses, such uniform to be renewed at the cost of the Council at suitable periods by this committee on the recommendation of the medical officer of health; further, that in all future appointments to the office of health visitor such officers be provided with and be required to wear similar uniform clothing during usual hours of duty.

### "HEROIN" AND THE MEDICAL PROFESSION.

In Mr. Hocking's paper, of which an abstract appeared in The Hospital recently (March 24, 1917, p. 497), the fact was mentioned that when aceto-morphine was first marketed in this country by a British firm the medical profession would not look at it. Later on, when freely pushed and advertised by the Germans under the disguise of the name

"heroin," it attained great popularity, a good part of which it still retains. In a vague sort of way most doctors are aware that "heroin" is a derivative of morphine, and cherish the belief that it possesses all the virtues and none of the drawbacks of that wonderful drug. But the course of time has shown that the "heroin" habit is just as easily acquired, just as difficult to shake off, and just as disastrous as the morphine habit; and that in other ways there is little to choose between the two. "Heroin," admittedly, has a beneficial effect on pain, and also upon some kinds of cough; but then morphine is equally efficacious. It is, therefore, with some interest that British physicians will note the views of representative American doctors, serving on the Committee on Drug Addiction in the United States, about heroin. It was resolved that "in the opinion of the Committee the drug heroin is of no real value in the practice of medicine, and that its place may be better taken by more efficacious agents that do not menace public welfare. Resolved that the Committee recommend Federal legislation to prevent the importation, manufacture, and sale of heroin in the United States of America."

### A MAGAZINE OF CURIOSITIES.

THE *Searchlight*, the monthly gazette of the 2nd Western Hospital, Manchester, differs from most other publications of its class in its odd and pleasant mingling of seriousness and chaff. The March number, which lies before us, for instance, contains an agreeable page by the editor almost in the light literary vein of Andrew Lang, headed, too, with a quotation from Keats; a page or two later the reader is instructed on the effect of diarrhœa in locusts, opposite which is a column of classic epigrams (mostly spiteful) on "Woman"; and the remaining contents include a series of quotations from the poets on "Drink." (Has its author read Mr C. L. Leipoldt's witty and learned chapter on the subject in "Commonsense Dietetics"?) Some sketches and light verse, and even a Bookman's Corner about Bacon? This medley is so agreeably mixed of diverse elements that a dull page is hard to find. We hope the editor will continue to be as catholic in his interests in future numbers. Perhaps the best example of such diversity is the elder Disraeli's "Curiosities of Literature," which will reward any editor who turns to it for suggestions, humour, and out-of-the-way information. It represents what journalism of this character can be at its best.

### A BIRMINGHAM DRAUGHTSMAN

THE patients and staff of the 1st Southern General Hospital, Birmingham, are fortunate in having secured for their gazette, the *Southern Cross*, a draughtsman with ideas in W. L. S. He has contributed to the March number a vivid portrait in black and white, and a remarkable frontispiece called "The Cloud," none the worse for being a symbol which readers must interpret as best they may. To give a lifted horizon is a more valuable service than merely to make bare statements of fact, and this is so often forgotten by draughtsmen that we welcome any drawing which is no mere illustration to a commonplace event. We hope W.L.S. will be encouraged, for his work has the place of honour that it deserves. The pseudo-Greek play is also a good thing, amusing without being banal. We like "Phormup" as the Greek equivalent for sergeant.

### A NEW NUMBER.

WE congratulate Colonel David Hepburn, commanding the 3rd Western General Hospital, Cardiff, for having been persuaded, doubtless by some instinctive journalist, to break the long silence which has concealed the doings of that institution for two and a-half years, by publishing at last a gazette, entitled the *Bulletin*. If this period had been devoted to collecting material, what a first number we should have had! As it is, draughtsmen, notably the graceful W. Leslie Elstow, versifiers, and contributors have suddenly appeared, and, with editorial encouragement, should soon get into their stride. Mr. Elsow has "arrived" with his first drawing. It was a good idea, since the hospital is housed in adapted buildings, to give some account of the voluntary agencies whose help made its creation possible, and the paragraphs "About those who have left us" might well be imitated in other gazettes. May they return in the form of contributions giving an account of their doings and sensations all over the world!

### THIS WEEK'S DRUG MARKET.

THE rising tendency in the prices of drugs continues to be the chief feature of the drug market, and far from there being any sign that this tendency is likely to receive a check, it seems more probable that it will become accentuated as time goes on. In the case of certain drugs which are now being produced in this country on an increasing scale, it is true that we may see somewhat lower prices, but many of the drugs for the supply of which—whether in the raw or the finished state—we depend on other countries will, in all probability, be still dearer. As a result of the absence of supplies of Epsom salts from the United States the price of this drug has advanced materially, and the tendency is still in an upward direction. Sarsaparilla, calumba root, balsam Peru, liquorice root, castor oil, and paraldehyde are among other drugs whose prices are advancing. Reports from Norway regarding the cod-fishing show that very slow progress is being made, and it seems certain that extravagant prices will continue to rule for cod-liver oil. There is still a shortage of cocaine, and high prices have to be paid for the alkaloid. As regards synthetic drugs the downward tendency in the price of aspirin continues, and supplies are plentiful; barbitone is extremely scarce and fetching extravagant prices; phenazone is becoming scarcer; the downward tendency in the price of phenacetin has come to a stop, as supplies are not coming in freely from abroad and British makers are not able to produce it in sufficient quantities to meet the large demand; amidopyrine is getting scarcer and dearer; guaiacol carbonate and resorcin are difficult to find, and famine prices are paid for anything available.

